# NUAK2 mediated regulation of Schwann Cell proliferation and migration in peripheral nerve injury via YAP

**DOI:** 10.1016/j.heliyon.2024.e34127

**Published:** 2024-07-04

**Authors:** Weidong Zhang, Yingchen Ni, Jianxin Li, Runjia Hua, Yudong Wang, Huilin Yang, Xuefeng Li, Minfeng Gan, Genglei Chu

**Affiliations:** a Department of Orthopaedic Surgery, The First Affiliated Hospital of Soochow University, Suzhou, Jiangsu, China; b Department of Orthopaedic Surgery, Affiliated Hospital of Nantong University, Nantong, Jiangsu, China; c Suzhou Medical College of Soochow University, Suzhou, Jiangsu, China

## Abstract

NUAK2 is a member of the AMP-activated protein kinase (AMPK) family, which plays an essential role in cellular processes such as apoptosis, proliferation, and cell fate. Recent studies have already shown that silencing of NUAK2 blocks proliferation and promotes apoptosis of human melanoma cells and liver cancer cells. In addition, NUAK2 is involved in the development of glioblastoma via regulating the expression of cancer stem cell-related genes, and it promotes the cell cycle entry in the glioblastoma cells. However, the expression and the role of NUAK2 in the progress of peripheral nerve regeneration after injury are yet to be elucidated. We observed that NUAK2 was upregulated following distal sciatic nerve crush (SNC). Interestingly, we discovered that NUAK2 showed co-localization with S100 (Schwann cell marker). Furthermore, we found that the NUAK2 had a spatiotemporal protein expression, which was consistent with proliferating cell nuclear-antigen (PCNA). The protein level of NUAK2 and YAP was upregulated in the model of TNF-α-induced Schwann cell (SC) proliferation. Furthermore, flow cytometry analysis, CCK-8, transwell assays, and wound healing assays were all performed with the purpose of exploring the role of NUAK2 in the regulation of SC proliferation and migration. More importantly, we found that NUAK2-deficient SCs showed significantly reduced expression of Yes-associated protein (YAP). Bioinformatic analysis identified upstream regulators of NUAK2 and NUAK2-associated genes (e.g., YAP1). Finally, we investigated the recovery changes during regeneration progress through the walking track analysis. Thus, we speculated that NUAK2 was involved in biochemical and physiological responses of SCs after SNC via YAP-driven proliferation and migration, and this study determined the importance of NUAK2 as a potential target in peripheral nerve regeneration.

## 1 Introduction

Peripheral nerve injuries (PNI) resulting from accidental lesions, ischemia, or deliberate surgery may lead to sensory disorder, autonomic nervous system dysfunction, and permanent movement disorders [1]. As opposed to the central nervous system (CNS), the peripheral nervous system (PNS) has an innate regenerative capacity. One of the most important reasons for the regenerative ability of the PNS is the surrounding Schwann cell (SC) and basal lamina, which provide physical support and nutrition to the axons to create a growth-permissive environment [2]. Following PNI, mature SCs undergo phenotypic modulation, and Wallerian degeneration occurs after nerve fiber is cut and distal axons are disrupted, followed by degradation of the myelin sheath and phagocytosis of the debris by both SCs and macrophages [3,4]. Following this, the denervated SCs respond to loss of axons by extrusion of their myelin sheaths, dedifferentiation, proliferation, and migration. The cells finally align with tubes and form the band of Bungner warp, which help to create a permissive microenvironment for regeneration of the PNS [5]. Considering the critical role played by SCs, investigation of the cellular and molecular mechanisms of SC proliferation and migration might help to promote repair and regeneration after PNI by providing a novel therapeutic target.

Neurite regeneration is a highly energy-dependent process, and AMP-activated protein kinase (AMPK) plays an essential role in controlling cellular nutrient and energy balance. NUAK2, also known as SNF1/AMP kinase-related kinase (SNARK), is a member of the AMPK family, which has been linked to the maintenance of cellular homeostasis [6,7]. NUAK Kinase roles in cortical axon arborization, with overexpression or downregulation of NUAK Kinase significantly increasing or reducing axon branching, respectively [8]. Alemany et al. demonstrated that mutation of NUAK Kinase have been linked to attention-hyperactivity/deficit and autism spectrum disorder. Cause defect of NUAK kinase can lead to the disorder of cortical connectivity, thus result in behavioral deficits such as social novelty, abnormal sensorimotor gating and spatial memory consolidation in mice [9]. Furthermore, NUAK2 has been reported to function in neural tube regeneration, with NUAK2 knockout mice display spina bifida, exencephaly, and facial clefting [10]. Since NUAK2 plays a significant role in cellular homeostasis, axon arborization, and neurodevelopmental disorders, it is important to further investigated the role of NUAK2 during all steps of neural tube regeneration, including peripheral nerve regeneration after injury.

Hippo-YAP pathway has been implicated in wound repair and regeneration [11]. Recent study indicated that YAP/TAZ regulate neurodevelopment, as nerves deficient for YAP exhibiting severe impairment of axon regrowth, Bunger band formation and remyelination [12]. It was recently demonstrated that NUAK2 is an essential mediator of YAP-driven cell growth, and knockdown of NUAK2 induces a decrease in migration and promotes cellular senescence of cancer cells with high YAP activity [13]. Besides, using both pharmaceutical and genetic inhibition of NUAK2, Yuan et al. provide evidence for its requirement in YAP-driven tumorigenesis and proliferation [14]. However, to date, the importance of NUAK2 in the pathology of PNI and regeneration has not been elucidated.

Based on the prominent effects of NUAK2 on nerve growth as well as the general dependency of SC proliferation on YAP activity. Here we use Sciatic Nerve Crush (SNC) model as a model system to elucidate crucial drivers of NUAK2 function. We induced SC proliferation with TNF-α at a low dosage and evaluated expressions of NUAK2 and YAP. Furthermore, by using genetic inhibition of NUAK2, we provide evidence for its requirement in YAP-driven migration and proliferation of SCs. Collectively, this study highlights the important role of NUAK2 in the PNS as well as the cellular and molecular mechanisms underlying SC during regeneration of the PNS.

## 2 Materials and methods

### 2.1 Animals and sciatic nerve crush model

Fifty-four male Sprague Dawley (SD) rats weighing 300 ± 50 g were acquired from the Animal Center of Soochow University (license No. SCXK [Su] 2021-0009). The Sciatic Nerve Crush (SNC) model was established as previously described [15]. The rats were deeply anesthetized with 10 % chloral hydrate (400–500 mg/kg), after that, all surgeries were performed in an aseptic environment. The sciatic nerve of rats was exposed and identified by blunt dissection. A small hemostat was used to crush the nerve at the mid-point for 10 s, and then it was unclamped for 10 s. This procedure was repeated 3 times. After all the procedures, the rats were allowed to recover in the cages. The rats were killed at various time points after sciatic nerve surgery. At the indicated time point, a 1-cm-long sciatic nerve segment centered on the lesion site was resected and treated with moderate penicillin powder until use.

### 2.2 Western blot analysis

Tissues were extracted from non-injured and injured sciatic nerve at the indicated time point after surgery and homogenized in lysis buffer (Thermo Scientific, Massachusetts, USA). SC samples were collected and lysed with extraction buffer (Sangon Biotech, Shanghai, China) containing PMSF, leupeptin, and aprotinin. Equal amounts of protein samples were subjected to SDS-PAGE and electro-transferred onto polyvinylidene difluoride filter membrane. Membranes were incubated with primary antibody for 24 h and then horseradish peroxidase-labeled secondary antibodies were used. Finally, membranes were scanned with chemiluminescence system (Bio-Rad). The information about primary antibodies is displayed as below. NUAK2 (1:500) was purchased from Abcam. Proliferating cell nuclear antigen (PCNA, 1:500) and p27Kip1 (1:100) were purchased from Santa Cruz Biotechnology. YAP (1:100) and GAPDH (1:1000) were purchased from Cell Signaling Technology.

### 2.3 Immunohistochemistry

Immunohistochemistry was performed as previously described [16]. Briefly, formalin-fixed paraffin-embedded sciatic nerve samples at different time points after surgery were cut into 5-μm sections for immunohistochemistry. Sections were blocked with confining liquid, followed by incubation with NUAK2 primary antibody (1:500; Abcam) for 24 h at 4 °C. NUAK2 antibody used for immunohistochemistry was already validated by siRNA in sciatic nerve and SC. Then sections were incubated with the biotinylated secondary antibody (Jackson ImmunoResearch Inc., PA, USA). Counterstaining was performed with hematoxylin. Sections were visualized by a light microscope (Zeiss Axiovert 200, Carl Zeiss Inc., NY, USA).

### 2.4 Immunofluorescence

All nerve sample sections were washed 3 times with PBS. Then the sections were blocked with confining liquid consisting of BSA (1 %), Triton X-100 (0.3 %), donkey serum (10 %), and Tween-20 (0.15 %) for 2 h. After that, the sections were incubated with primary antibodies for 24 h at 4 °C, followed by incubation in a mixture of TRITC- and FITC-conjugated secondary antibodies for 2 h at room temperature. NUAK2 (1:500) and NF200 (neuronal marker, 1:300) were purchased from Abcam, and S100 (SC marker, 1:100) was purchased from Sigma-Aldrich. The total number of cells was counted along with the number of positive cells in each corresponding stain. Analysis was done using ImageJ Software (http://imagej.nih.gov/ij).

### 2.5 Cell culture

SCs obtained from the sciatic nerve of neonatal SD rats were isolated as described previously [17]. Sciatic nerves were shredded, and then they were dissociated by mixing in phosphate-buffered saline containing trypsin (0.25 %, Sigma) and collagenase A (0.1 %, Sigma). Then, cells were cultured in Dulbecco's modified complete medium (Invitrogen) on a poly-l-lysine-coated culture dish for one day in an incubator and allowed to adhere. Non-SCs were then eliminated after being treated with cytosine arabinoside (10 μM, Santa Cruz) three times, followed by subjection to immunopanning with an antibody against THY1.1 (Santa Cruz). The isolated SCs were treated with 2.0 ng/ml TNF-α (Sigma) for proliferation experiments.

### 2.6 Vectors and transfection

NUAK2 short-interfering RNA (siRNA) oligos and an empty vector (provided by Sangon Biotech of Shanghai) were used. In order to avoid the non-specific effects on other genes, two siRNA sequence against NUAK2 (si-NUAK2-1/si-NUAK2-2) and a nontargeting siRNA used as a negative control. The targeting sequence of NUAK2 siRNA was as follows: si-NUAK2-1, 5′-ACCATAAGATCCTAGTGAA-3’; si-NUAK2-2, 5′-ATCTCAGACCCCTTCTGCA-3’. SCs were transfected using Lipofectamine 2000 (ThermoFisher, CA, USA) as suggested in the protocol.

### 2.7 Bioinformatic analysis

StarBase (https://rnasysu.com/encori/), miRWalk (http://mirwalk.umm.uni-heidelberg.de/), TargetScan (https://www.targetscan.org/vert_80/), and miRDB (https://mirdb.org/cgi-bin/search.cgi) were used to predict potential upstream miRNAs of NUAK2. The online Venn tool was used to filter out anticipated upstream miRNAs that overlapped. STRING (https://cn.string-db.org/) and Cytoscape software v3.10.0 (http://cytoscape.org/) was applied to identify NUAK2-associated genes.

### 2.8 CCK-8 assay

SCs were transfected with siRNA overnight, and then they were seeded onto 96-well cell culture cluster plates at a concentration of 2 × 10^4^ cells/well. After overnight incubation, 10 μl Cell Counting Kit-8 (CCK-8; Enzo Life Sciences) reagents were added to each well. After incubation, the absorbance was measured using a microplate reader at 450 nm.

### 2.9 Flow cytometry analysis

Cells were harvested and washed with PBS three times, and then they were fixed in cold 70 % ethanol for 30 min at 4 °C. After that, the cells were incubated with ribonuclease by adding RNase A (1 mg/ml) for 30 min at 37 °C. Subsequently, the cells were stained with 50 mg/ml propidium iodide (PI, Abcam) in PBS, and then they were analyzed by flow cytometric analysis apparatus (Guava® easyCyte, Merck Millipore, Germany).

### 2.10 Cell migration assay

A modified Boyden chamber assay was used to investigate the effect of NUAK2-siRNA on SC migration. Two days after transfection, 5 × 10^4^ cells/well were starved for 12 h, and they were placed on the upper layer of each transwell. SCs were allowed to migrate into the lower chamber which consisted of complete medium. Following an incubation period, cells that had migrated through the membrane were fixed with 4 % paraformaldehyde and stained with toluidine blue, and the number of cells was counted as qualified migration on each filter.

### 2.11 Wound healing assay

SCs transduced with NUAK2-siRNA were seeded on a 6-well plate in DMEM supplemented with 10 % FBS for 24 h growth, and they were allowed to grow to 70 %–80 % confluence. After that, the monolayer was slowly scratched with a new 1 ml pipette tip across the center of the well. After scratching, the cells were gently washed twice with medium to remove the detached cells, and the well was replenished with serum-free medium. Photographs of the cells were taken after wounding at the indicated time points.

### 2.12 Walking track analysis and open field tests

The LV-NUAK2 lentivirus was obtained from GenePharma technologies Co., Ltd. At the time the sciatic nerves were crushed, 10 μl vector or LV-NUAK2 were injected into the proximal nerve conduits. Mouse paw prints were captured by applying red ink to their hind paws and allowing them to walk down a corridor measuring 6 cm by 40 cm, which was lined with white paper. These prints were then scanned, and measurements of toe spread and paw length were taken. The sciatic functional index (SFI) was determined using a previously established formula: SFI = 118.9 (ETS− NTS)/NTS−51.2(EPL−NPL)/NPL−7.5 [18], where ETS represents the experimental toe spread, NTS the normal toe spread, EPL the experimental paw length, and NPL the normal paw length. An SFI close to zero signifies normal nerve function, while a score around −100 indicates complete impairment.

The Open Field test, a straightforward sensorimotor assessment, is utilized to evaluate general activity levels, gross motor skills, and exploratory behaviors in rodent models with peripheral nerve injury (PNI) disorders. This evaluation involves placing the animal in a square, white Plexiglas box and allowing it to move freely for 10 min. An overhead camera records the activity, and an automated tracking system analyzes the data for movement distance, velocity, and time spent in specified zones.

### 2.13 Statistical analysis

Data are shown as mean ± SEM. *P < 0.01 or *P < 0.05 was considered to indicate a significant difference. Statistical analysis was performed by one-way analysis of variance (ANOVA) with Tukey's post-hoc multiple comparison tests. Quantifications were performed for at least three replicates per condition.

## 3 Results

### 3.1 Changes in protein expression and cellular localization of NUAK2 in the SNC model

**Fig. 1:**
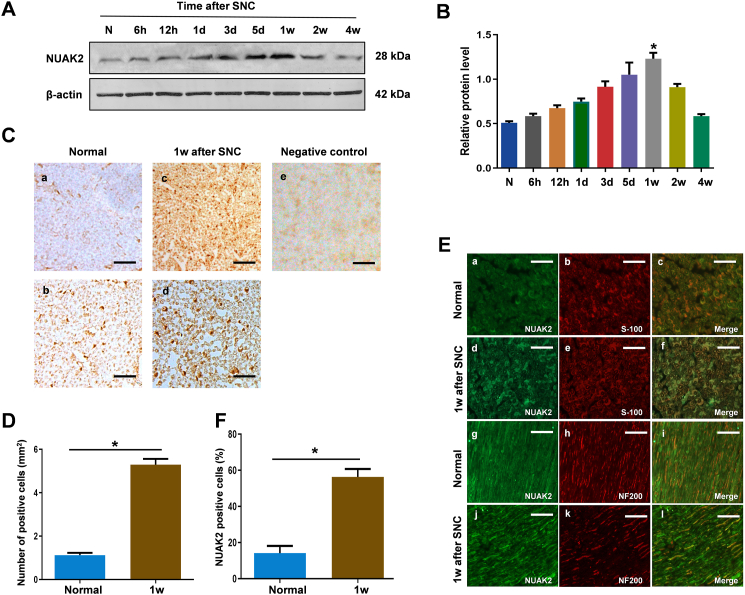
Expression and location of NUAK2 changes following SNC. **A** Western blot analysis of NUAK2 at the indicated times. **B** Relative protein expression level of NUAK2 was quantitated by densitometry. β-actin level was used as a control. Data are presented as mean ± SEM; n = 3. **C***a-e* Immunohistochemical detection of NUAK2 in sham (***a***) and SNC groups (***c***) on low magnification views. Higher magnification views are shown for sham (***b***) and SNC groups (***d***). Negative control (***e***). *Scale bars*, 100 μm (***a, c, e***) and 40 μm (***b, d***). **D** Quantitative analysis of NUAK2-positive cells/mm^2^ in normal rats and at 1 w after SNC. Data are presented as mean ± SEM; n = 20. **E** Double immunoﬂuorescence staining of NUAK2 and different phenotype-speciﬁc markers, S100 (red, ***a-f***) and NF200 (***g-l***). *Scale bar*, 50 μm**. F** Quantitative analysis of S100-positive cells expressing NUAK2 in normal and SNC groups. Data are presented as mean ± SEM; n = 200 total cells (*p < 0.05 versus the normal group).

To ascertain the expression pattern and changes in the distribution of NUAK2 in the SNC model, Western blot, immunostaining, and immunohistochemistry staining were performed in the normal and SNC groups at the indicated time points. Western blot showed that NUAK2 expression was markedly increased after 3 days post-injury, and it peaked at 1 w and decreased to the normal level at about 4 w (Fig. 1A and B). The NUAK2 level was counted in the normal and SNC groups by immunohistochemistry staining. The percentage of NUAK2-positive cells was observably increased compared with that in the normal sciatic nerve (Fig. 1C). Quantitative analysis results of NUAK2-positive cells after SNC were consistent with Western blot results for NUAK2 expression after SNC (Fig. 1D). Meanwhile, we performed double immunofluorescent staining in transverse and longitudinal cryosections of the sciatic nerve at 1 w post-injury. Notably, compared with the normal group (Fig. 1E a−c), the expression of NUAK2 was significantly increased in SCs (Fig. 1E d−f) at one week after SNC, while the expression patterns of NUAK2 and NF200 barely showed co-localization in the axons of both normal (Fig. 1E g−i) and SNC groups (Fig. 1E j−l). Quantitative analysis results indicated that the expression of NUAK2 was significantly increased only in SCs after SNC, compared with that in the normal sciatic nerve (Fig. 1F).

### 3.2 Correlation of NUAK2 expression with SC proliferation following SNC

**Fig. 2:**
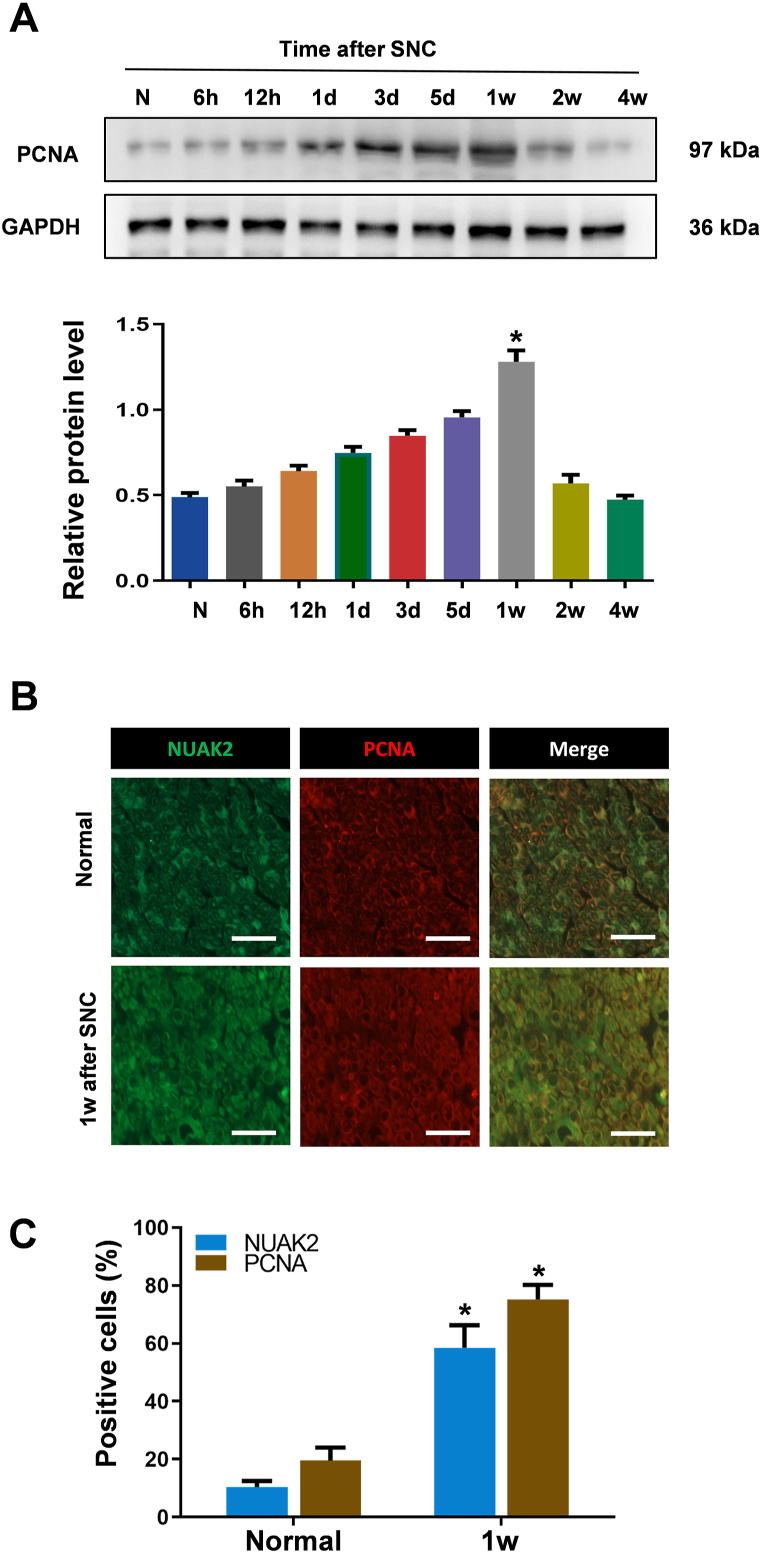
Expression changes in NUAK2 following SNC. A Western blot analysis of PCNA at the indicated times after SNC. GAPDH was used as a control. Relative protein expression level of NUAK2 was quantitated by densitometry. GAPDH level was used as a control; *n* = 6 rats per time point. **B** Double immunofluorescence staining for NUAK2 (green) and PCNA (red) at 1 w after surgery. **C** The positive cells of NUAK2 and PCNA was quantitated. *n* = 200 total cells, *p < 0.05 versus the normal group. *Scale bar*, 50 μm.

SCs respond to PNI by cellular reprogramming that generates cells specialized for promoting repair and regeneration, although SCs injury response is central to nerve repair, the nature of this process has been uncertain and disputed. Since it was found that NUAK2 modulated glioma proliferation by inhibiting p27^kip1^ [19,20], we detected the expression pattern of PCNA (a general marker of dividing cells) to determine the relationship between NUAK2 and the proliferation of SCs. As shown in Fig. 2, we found that the expression of PCNA was increased at 1 w post-injury (Fig. 2A and B), which was similar to the increase in the protein level of NUAK2. Double immunofluorescent labeling showed that NUAK2 showed higher co-localization with PCNA at 1 w after SNC compared with that in the normal group (Fig. 2C). Together, these findings indicated that the increase in NUAK2 might be associated with SC proliferation after SNC.

### 3.3 Effects of NUAK2 in the TNF-α induced SC proliferation mode

**Fig. 3:**
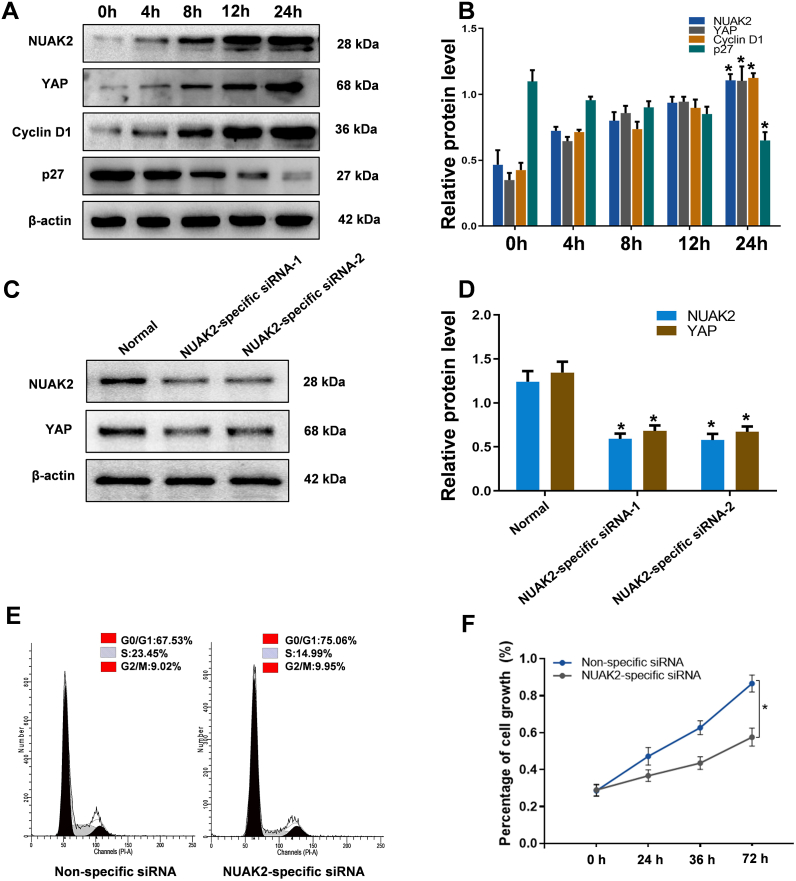
Effects of NUAK2 on SC proliferation. **A** Western blot analysis of NUAK2, YAP, Cyclin D1, and p27 in SCs after TNF-α treatment at the indicated times. **B** Quantitative graphs revealed enhanced expression levels of NUAK2, YAP and Cyclin D1 and decreased expression of p27 in SCs after treatment. β-actin level was used as a control. Data are presented as mean ± SEM; n = 3. **C** Western blot analysis demonstrated that NUAK2-siRNA-1 and NUAK2-siRNA-2 markedly decreased the NUAK2 and YAP levels in SCs. **D** Bar chart showing the ratio of NUAK2 and YAP to β-actin. Data are presented as mean ± SEM; n = 3. **E** The cell cycle phases of transfected SCs were as described above, and they were analyzed by flow cytometry. **F** Cell proliferation was measured by the CCK-8 assay. NUAK2 siRNA-transfected SCs showed significant inhibition of cell proliferation. n = 6, *p < 0.05 indicates significant differences compared with the control group.

To further explore the function of NUAK2 in SCs proliferation, we treated the cells with low dosage of TNF-α [21]. As shown by Western blot, the expression levels of NUAK2, YAP, and Cyclin D1 were increased during the process of TNF-α induced SC proliferation, while the expression level of p27 expression was decreased (Fig. 3A and B). The results demonstrated that NUAK2 was related to SC proliferation by amplifying YAP activity. To further validate the hypothesis, we suppressed NUAK2 expression with NUAK2-specific small oligo-siRNA. The efficiency of NUAK2-siRNA was analyzed by Western blot. Results showed that both NUAK2-siRNA-1 and NUAK2-siRNA-2 could reduce the protein levels of NUAK2 in SCs. In addition, we observed decreased expression levels of YAP in NUAK2-siRNA-1 and NUAK2-siRNA-2 transfected cells (Fig. 3C and D). As NUAK2-siRNA-1 was more efficient. Thus, NUAK2-siRNA-1 was selected to knock down NUAK2 expression. Cell cycle distribution detected by flow cytometry showed accumulation of SCs in the G0/G1 phase in NUAK2-specific siRNA-transfected cells as compared with the nonspecific control (Fig. 3E). Moreover, CCK-8 assays also showed that NUAK2 depletion decreased the cell growth rate compared with that in the control (Fig. 3F). According to the results, depleting the expression of NUAK2 could suppress the G1/S-phase transition of SCs and impede the proliferation of SCs compared with those in the nonspecific control. Therefore, these data confirmed that NUAK2 was involved in the regulation of SC proliferation.

### 3.4 Effects of NUAK2-specific siRNA on SC migration

**Fig. 4:**
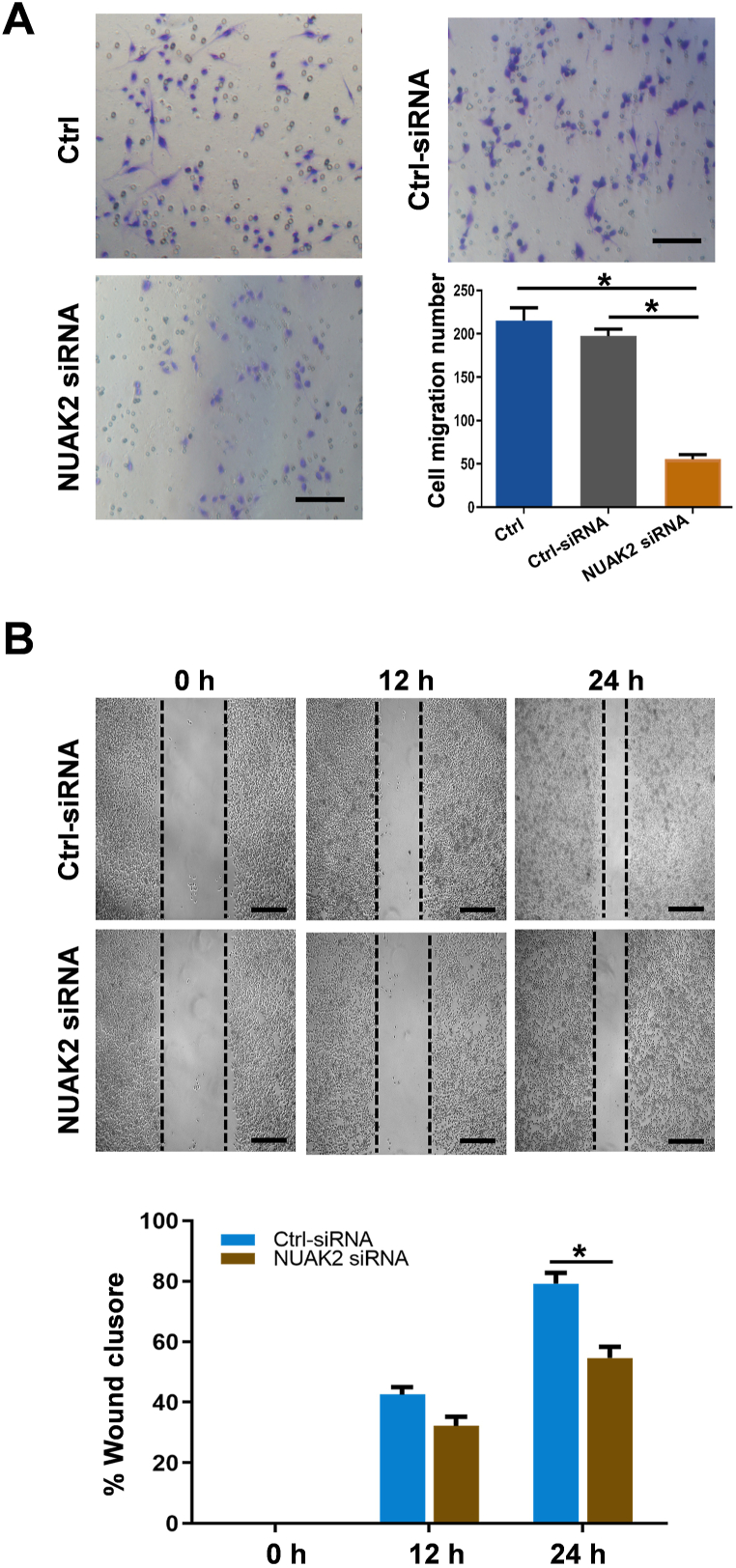
Effects of NUAK2-specific siRNA on cell migration. **A** Transwell assays of normal and NUAK2-specific siRNA-transfected SCs. The data are expressed as mean ± SEM (n = 3; *p < 0.05) *Scale bar*, 50 μm**. B** Wound healing assays for the non-specific and NUAK2-specific groups. *Scale bar*, 100 μm. Quantitative analysis of two groups. Data are presented as mean ± SEM (n = 3, *p* < 0.05).

SC migration plays a critical role in promoting the regeneration of peripheral nerve axons and in replacing the lost myelin [22]. Therefore, cell migration and wound healing assays were performed in NUAK2-specific siRNA-transfected SCs. We found that NUAK2-specific siRNA-transfected SCs showed decreased migration compared with normal or nonspecific siRNA-transfected SCs (Fig. 4A and B). These results demonstrated that NUAK2 was involved in SC migration.

### 3.5 Effects of LV-NUAK2 on functional recovery of SNC in rats

**Fig. 5:**
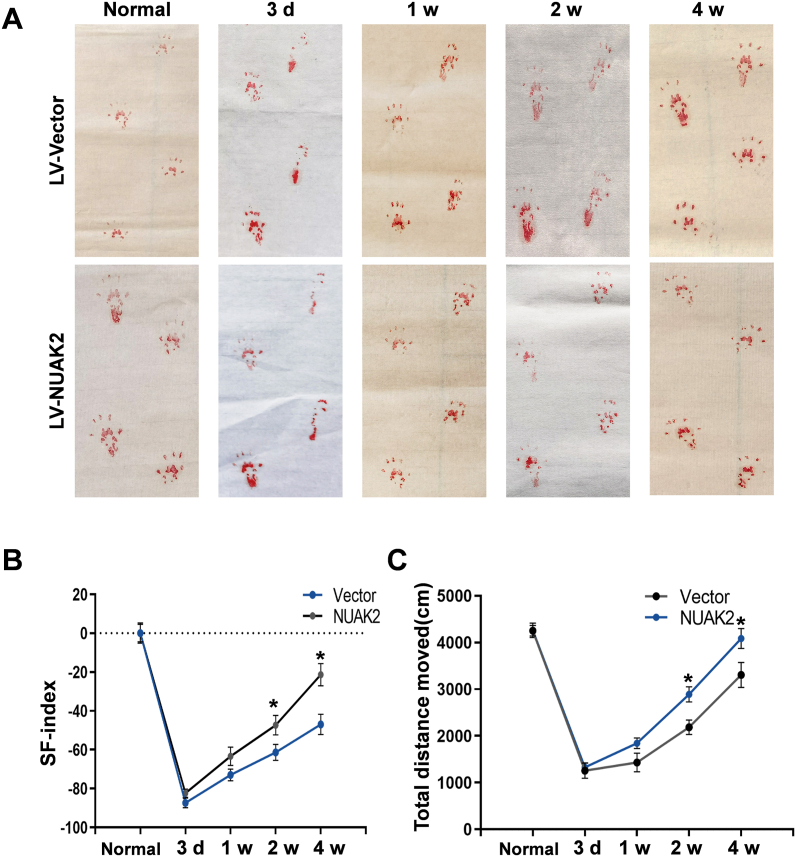
Effects of LV-NUAK2 on the functional recovery after SNC. **A** Footprints of the rats treated with LV-Vector or LV-NUAK2. **B** Comparison of functional recovery determined by SFI. Data are presented as mean ± SEM; n = 6. **C** Comparison of functional recovery determined by Total distance moved. Data are presented as mean ± SEM; n = 6.

After a microinjection of 10 μl LV-NUAK2 viral into SNC rats, the SFI value and total distance moved characterized crucial aspects of locomotion activities involving recovery of motor function and hindlimb sensory. The SNC group exhibited reduced SFI levels, while the LV-NUAK2 group represented wrought-up recovery changes during the regeneration progress (Fig. 5A–C). We speculate that LV-NUAK2 advanced the process of regeneration via promoting the proliferation of SCs after SNC.

### 3.6 Construction of NUAK2-centered genetic network

**Fig. 6:**
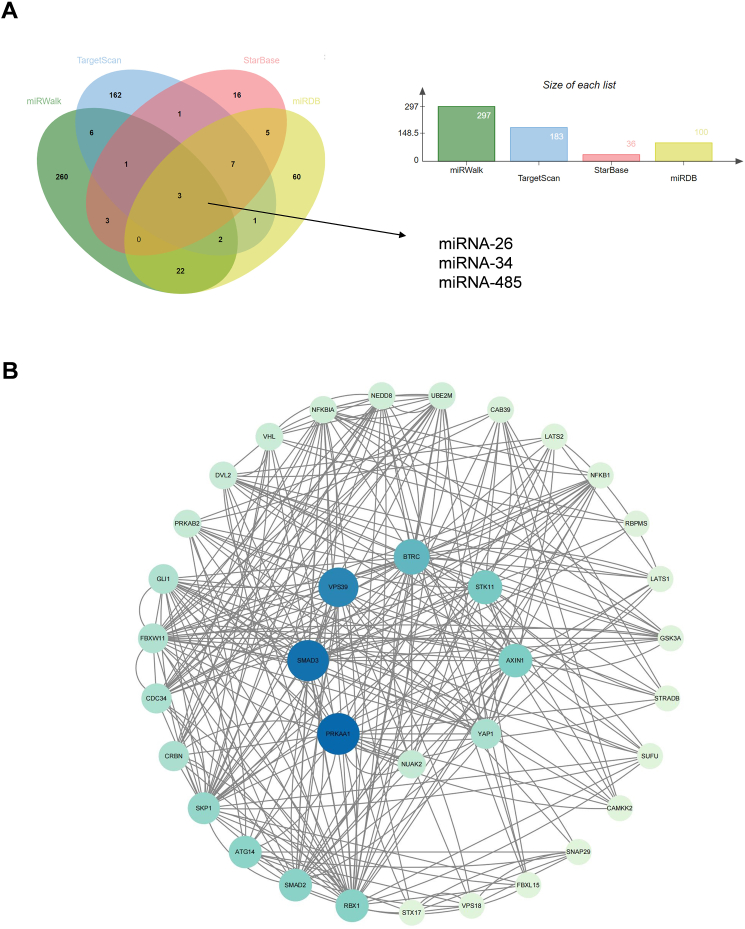
Bioinformatic analysis of NUAK2-related genes. **A** Venn diagram of upstream miRNA of NUAK2 predicted by miRWalk, TargetScan, StarBase and miRDB. **B** The genetic network of NUAK2-associated genes.

The bioinformatic tools as miRWalk, TargetScan, StarBase and miRDB were applied to determine upstream factors and downstream regulators of NUAK2. The miRNA-mRNA prediction software miRWalk, TargetScan, StarBase and miRDB screened 297, 187, 36, and 100 miRNA as candidate upstream regulatory miRNAs of NUAK2, respectively. Joint analysis of these prediction software indicated that miRNA-26, miRNA-34, and miRNA-485 might be upstream regulatory miRNAs of NUAK2 (Fig. 6A).

Target genes of NUAK2 were also identified using STRING and Cytoscape software. A total of 34 genes, including Yes1 associated transcriptional regulator (YAP1), AXIN1, serine/threonine kinase 11 (STK11), beta-transducin repeat containing E3 ubiquitin protein ligase (BTRC), VPS39 subunit of HOPS complex (VPS39), SMAD family member 3 (SMAD3), and protein kinase AMP-activated catalytic subunit alpha 1 (PRKAA1) were identified to be associated with NUAK2 (Fig. 6B).

## 4 Discussion

In this study, we established SNC rat model and characterized the role and cellular mechanisms of NUAK2 during PNI and regeneration. We investigated the expression of NUAK2 in rat sciatic nerves following injury and discovered that NUAK2 is related to reactive SCs. *In vitro*, double immunofluorescence staining demonstrated that the spatiotemporal expression of NUAK2 is parallel to that of PCNA, a proliferation marker, which insinuates that NUAK2 might have an association with the proliferation of SCs. *In vivo*, our results showed that NUAK2 is up-regulated from an early stage, and it peaks at 1 w and then gradually decreases to the normal level. Immunostaining revealed that the up-regulation of NUAK2 was predominant in SCs during PNI. Walking track analysis also provided preliminary evidence that NUAK2 might play a positive role in promoting functional rehabilitation after SNC.

As a member of the AMPK-related kinase family, NUAK2 resides in the region spanning lq31-lq32. Public databases showed that NUAK2 is highly expressed in a range of human cancers such as melanoma, glioblastoma, or other cancers; thus, suggesting that it might be associated with tumor progression and cancer development [[23], [24], [25]]. Recent studies have also demonstrated that NUAK2 is an emerging oncogene that regulates cell migration and cycle progression in eukaryotes [26,27]. However, limited information is available regarding the target of this kinase, and it is likely that other substrates could potentially contribute to oncogenesis downstream of NUAK2 [28]. Recently, some studies have indicated that NUAK2 enhancement is linked to unfolded protein response (UPR), which associated to regeneration and functional recovery after PNI [29,30]. NUAK2 localizes in the nucleus and cytoplasm of cells, and it contributes to various signaling pathways such as P27^Kip1^ (p27), mTOR, and YAP [[31], [32], [33]]. P27 may interact with a large range of cyclin-CDK complexes, and it blocks their catalytic activity, which plays a critical role in the control of S-phase population in cell cycle profile and cell proliferation. Several studies have indicated that p27 plays a significant role in controlling SC proliferation by inhibiting the transition of G1/S during PNI and repair [[34], [35], [36]]. It was reported that mTOR activation and CDK induction are connected with the expression of NUAK2, although the underlying mechanisms of how NUAK2 acts downstream remain to be determined [37,38]. In both glioblastoma and cancer stem cells, downregulation of NUAK2 by micro-RNA could inhibit PI3k; thus, leading to downregulation of cyclin D1 and upregulation of p27 [39,40]. Recently, Vallenius et al. demonstrated that NUAK2 levels are strongly induced by stimuli increasing actomyosin fiber formation, and NUAK2 is required for fiber maintenance in exponentially growing cells, indicating NUAK2 in a positive-feedback loop regulating actin stress fibers [41]. Besides, Yuan et al. provided multiple lines of evidence that the inhibition of NUAK2 represents a novel approach to modulate YAP function in the biological functions of cancer cells. NUAK2 acts as a central player in promoting the growth of liver tumors by targeting Hippo/YAP signaling [42]. Moreover, in human melanoma, knockdown of NUAK2 decreases the migration and induces cellular senescence of melanoma cells by regulating the expression of N-cadherin, a cell transmembrane protein, and function to mediate YAP-dependent cell−cell adhesion [43]. As a consequence, NUAK2 has profound effects on the proliferation and migration of cancer cells; and its downstream genes, including p27 and YAP, play a critical role in the regulation of cell division cycle and motility.

On the basis of the tumorigenic role of NUAK2 in the growth and migration of several human cancer cells, current researchers have explored the NUAK2-mediated regulation of cellular physiology in the nervous system. In the study of CNS injuries, Ohtake et al. demonstrated the key role of LKB1-NUAK1 signaling in improving the regenerative capacity of the CNS, which leads to significantly enhanced recovery by regulating the proliferation and differentiation of neurons [44]. Besides, AMPK signaling has long been regarded as an important metabolic regulator in SC control of axon integrity and myelination, and recently, several downstream AMPKs have been implicated in the maintenance of metabolic homeostasis in SC proliferation and migration [45]. Here, we aimed to investigate the role of NUAK2 in orchestrating the SC response to PNI.

During the recovery process, following the early stages of PNI, the SCs dedifferentiate, and the regulation of SC proliferation and migration might be indispensable. SC proliferation is dependent on an ordered cell cycle progression, which can be regulated by some CDKs, cyclin proteins, and CDKIs after PNI [34,46]. Previous studies have shown that the expression of p27 was decreased after PNI, and then it recovered to the normal level in about two weeks, which also indicated that p27 may be a negative regulator of peripheral nerve regeneration [47,48]. Hence, exploration of the detailed mechanism that controls the expression of p27 may be crucial for nerve repair. Recently, numerous studies have demonstrated that targeting YAP has become an intriguing avenue for cancer therapeutics as it is a key regulator of cell cycle progression [42,49]. Recent studies have also indicated that YAP/TAZ are required for peripheral myelination and radial sorting through Gαs-mediated SC proliferation and migration [12,[50], [51], [52]]. Our results demonstrated that in the model of TNF-α-induced SC proliferation, the expression levels of NUAK2, YAP, and Cyclin D1 were up-regulated, while p27 expression was distinctly down-regulated. NUAK2 depletion augmented the G0/G1 arrest and inhibited the YAP-dependent proliferation of SCs. Bioinformatic analysis also identified NUAK2-associated genes, including YAP1, VPS39, SMAD3, and PRKAA1. On combining these results with those of the experiments *in vivo*, our data provide preliminary evidence that NUAK2 participates in a particularly advancing effect on cell cycle progression and SC proliferation by affecting the YAP activation. Besides, NUAK2 depletion also results in reducing the migration of SCs, a phenomenon that might be related to reduced YAP activation. Recent studies have demonstrated that the lack of YAP and TAZ in stem cells during their developmental process leads to a significant peripheral neuropathy resulting from defects of axons [53]. The effects of TAZ loss in SCs upon nerve regeneration and whether loss of TAZ function will revert the peripheral nerve after injury remain to be tested.

Non-coding RNAs can regulate the expression of target genes and be applied as therapeutic methods. MiRNA-26, miRNA-34, and miRNA-485 were screened and selected as candidate regulatory miRNAs of NUAK2 by using bio-prediction tolls. Expression profiles of mouse sciatic nerve crush model showed that inhibition of miRNA-26 reduce axon regeneration by targeting GSK3 [54]. In addition, the overexpression of miRNA-34 evidently inhibited the migration and proliferation of SCs, and further study has demonstrated that miRNA-34 contributes to apoptosis and autophagy via the Hippo-YAP signaling pathway [55]. A study found that miR-485 overexpression targets rac1 and Cdc42 to inhibit the myelination and proliferation of SCs. Although the direct function of miR-485 in Schwann cells remains unknown, a recent study has demonstrated that miR-485 suppresses YAP1 expression through binding to the 3′UTR of its mRNA [56]. The beneficial roles of miRNA-26, miRNA-34, and miRNA-485, together with the promocccting role of NUAK2 siRNA on SCs plasticity, suggests that these potential miRNAs may regulate PNI repair and regeneration through suppressing and targeting NUAK2 regulated YAP expression.

## 5 Conclusion

The present study showed that NUAK2 was upregulated in SCs after SNC; thus, suggesting that NUAK2 is involved in regulating SC proliferation and migration after this type of injury. Further studies should aim at investigating the exact mechanism between NUAK2 and YAP/TAZ to promote myelination in the PNS after sciatic nerve injury.

## Data availability statement

The materials supporting the evidence reported in this article are included within the article. Data included in article/supplementary material/referenced in article.

## Funding

The study is supported by 10.13039/501100001809National Natural Science Foundation of China (32101103), the 10.13039/501100004608Natural Science Foundation of Jiangsu Province (BK20200199), 10.13039/501100002858China Postdoctoral Science Foundation (2023M742550, 2021M702412), Suzhou Medical Innovation & Application Program (SKY2022126).

## Ethics declarations

The study was approved by Medical Ethics Committee of The First Affiliated Hospital of Soochow University (ethical code 2020228).

## CRediT authorship contribution statement

**Weidong Zhang:** Data curation, Conceptualization. **Yingchen Ni:** Investigation, Data curation, Conceptualization. **Jianxin Li:** Resources, Data curation, Conceptualization. **Runjia Hua:** Visualization, Validation, Conceptualization. **Yudong Wang:** Writing – review & editing. **Huilin Yang:** Writing – original draft, Data curation, Conceptualization. **Xuefeng Li:** Writing – review & editing, Writing – original draft, Supervision. **Minfeng Gan:** Writing – review & editing, Writing – original draft, Validation, Supervision. **Genglei Chu:** Writing – review & editing, Writing – original draft, Visualization, Validation, Supervision, Software, Resources, Project administration, Methodology, Investigation, Funding acquisition, Formal analysis, Data curation, Conceptualization.

## Declaration of competing interest

The authors declare that they have no known competing financial interests or personal relationships that could have appeared to influence the work reported in this paper.
