# Supplementary material for: NUAK2 mediated regulation of Schwann Cell proliferation and migration in peripheral nerve injury via YAP
Source: Heliyon. 2024 Jul 4;10(13):e34127. doi: 10.1016/j.heliyon.2024.e34127 (PMC11282989; doi:10.1016/j.heliyon.2024.e34127)
Supplement: Multimedia component 1 [file mmc1.docx]

**Figure 1.**

**(A)** Expression of NUAK2 changes following SNC. Western blot analysis of NUAK2 at 6 h, 12 h, 1 d, 3 d, 5 d, 1 w, 2 w, 4w. β-actin was used as a control.


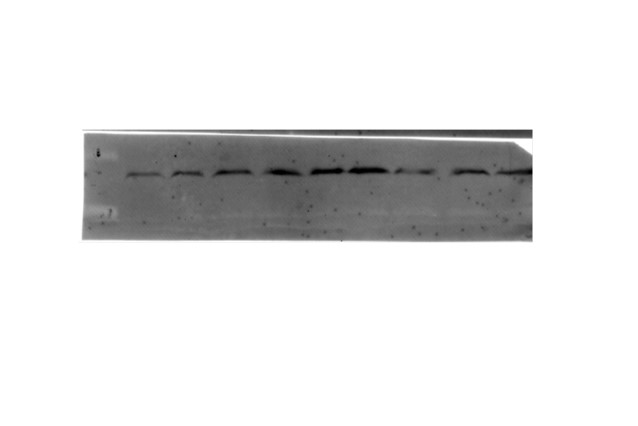

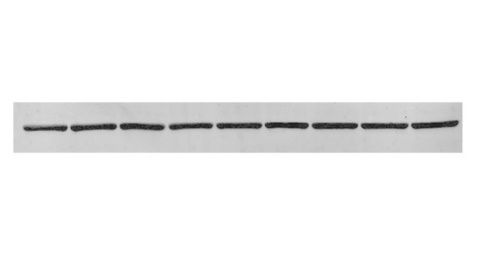


NUAK2 β-actin

**Figure 2.**

**(A)** Expression changes in PCNA following SNC. Western blot analysis of PCNA at the indicated times after SNC. GAPDH was used as a control.


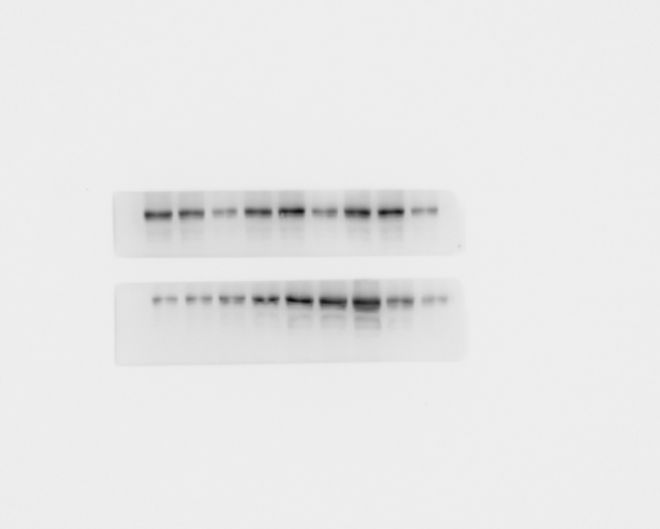

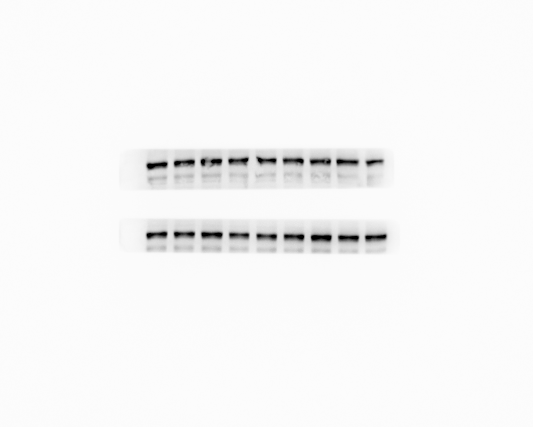


PCNA (the second stripe) GAPDH (the second stripe)

**Figure 3.**

**(A)** Effects of NUAK2 on SC proliferation. Western blot analysis of NUAK2, YAP, Cyclin D1, and p27 in SCs after TNF-α treatment at the 6 h, 12 h, 1 d, 3 d, 5 d, 1 w, 2 w, 4w.


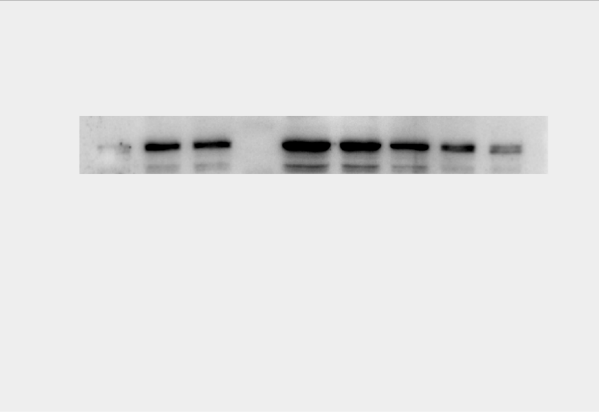

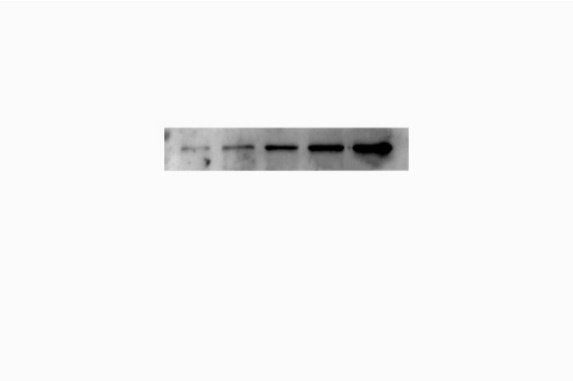


NUAK2 YAP


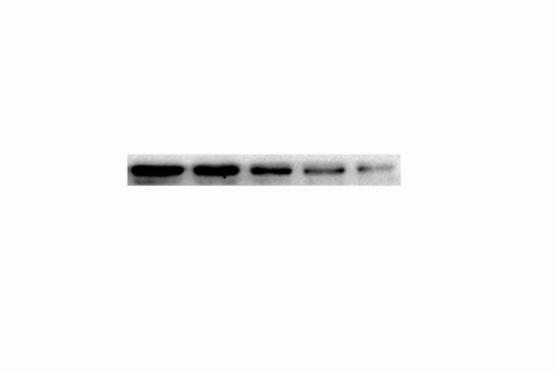

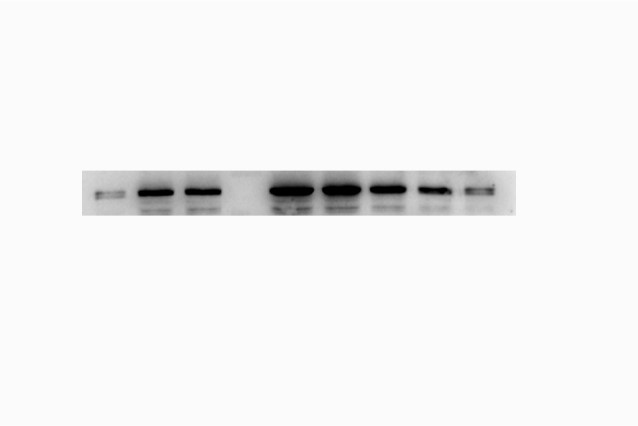


Cyclin D p27


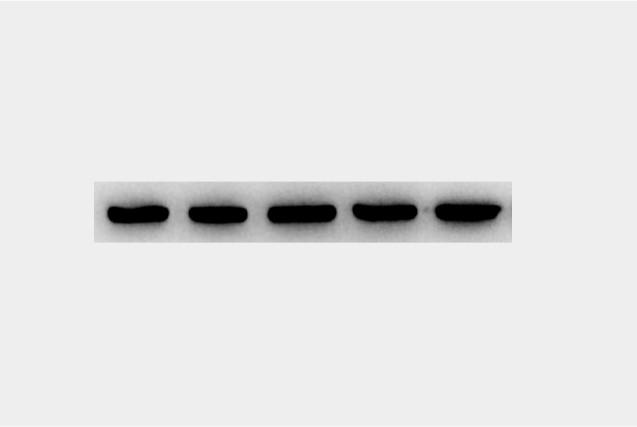


β-actin

**Figure 3.**

**(C)** Western blot analysis of NUAK2 and YAP after treatment of NUAK2-siRNA-1 and NUAK2-siRNA-2.


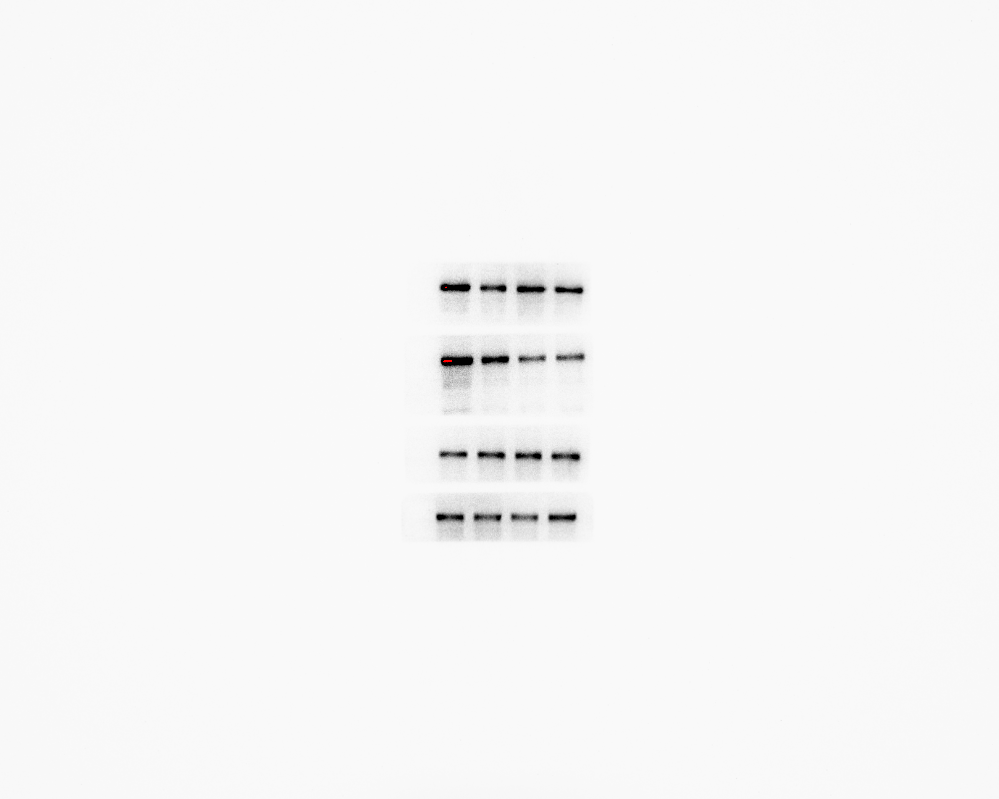


NUAK2 (the second stripe) and YAP (the fourth stripe)


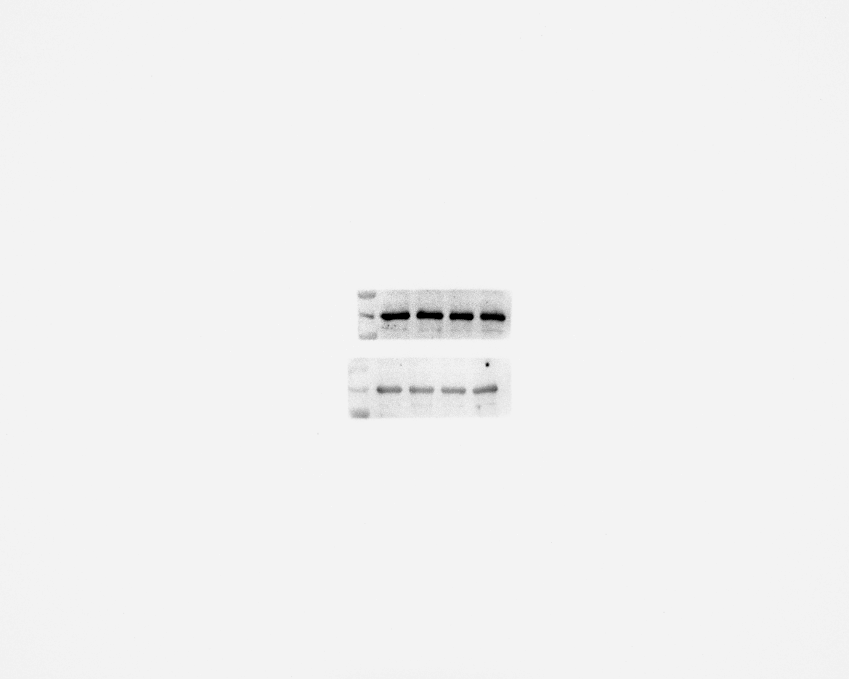


β-actin (the first stripe)
